# Supplementary material for: Authentication of smart grid communications using quantum key distribution
Source: Sci Rep. 2022 Jul 26;12:12731. doi: 10.1038/s41598-022-16090-w (PMC9325752; doi:10.1038/s41598-022-16090-w)
Supplement: Supplementary file 1 — Supplementary Information. [file 41598_2022_16090_MOESM1_ESM.pdf]

Authentication of Smart Grid Communications using Quantum Key Distribution: Supplementary Information

Muneer Alshowkan[1,*], Philip G. Evans[1], Michael Starke[2], Duncan Earl[3], and Nicholas A. Peters[1]

[1]Computational Sciences and Engineering Division, Oak Ridge National Laboratory, Oak Ridge, TN 37831, USA
[2]Electrification and Energy Infrastructures Division, Oak Ridge National Laboratory, Oak Ridge, TN 37831, USA
[3]Qubitekk Inc., Vista, CA 92081, USA
[*]alshowkanm@ornl.gov

Supplementary Information

Algorithm 1 Create MAC for each outgoing message

Input message, topic
Output payload

```
function PUBLISH(message)
    m ← message
    t ← topic
    n ← number of next secret key
    ts ← timestamp
    tm ← m + t + n + ts
    key ← the nth secret key from key table
    iv ← next random number from QRNG
    mac_S ← GMAC_E(tm, key, iv)
    p ← tm + iv + mac_S
    return p
end function
```

Algorithm 2 Verify MAC for each incoming message

```
    Input payload, topic
    Output True/False
function ON_MESSAGE(payload, topic)
    for p in payload do
        tm ← p[1]
        iv ← p[2]
        mac_s ← p[3]
    end for
    for q in tm do
        m ← q[1]
        t ← q[2]
        n ← q[3]
        ts ← q[4]
    end for
    ct ← current time
    key ← the nth secret key from key table
    mac_R ← GMAC_D(tm, key, iv)
    if mac_S = mac_R & t = topic & ts < ct − δ then
        result ← True
    else
        result ← False
    end if
    return result
end function
```
